# Indoor Air Quality in Tujia Dwellings in Hunan, China: Field Tests, Numerical Simulations, and Mitigation Strategies

**DOI:** 10.3390/ijerph19148396

**Published:** 2022-07-09

**Authors:** Fupeng Zhang, Lei Shi, Simian Liu, Jiaqi Shi, Mengfei Cheng

**Affiliations:** 1 School of Architecture and Art, Central South University, Changsha 410075, China; 201301004@csu.edu.cn (F.Z.); shilei@csu.edu.cn (L.S.); 191311004@csu.edu.cn (M.C.); 2 Health Building Research Center, Central South University, Changsha 410075, China; 3 College of Architecture, Changsha University of Science & Technology, Changsha 410114, China

**Keywords:** indoor air quality, firepit emissions, pollutant-dispersion simulation, passive mitigation strategies, Hunan Tujia region

## Abstract

Air pollution is a major health hazard. The traditional habits and unique ethnic fire culture in Hunan Tujia region result in the long-term exposure of residents, especially elderly people, to pollutants. In this study, we conducted field monitoring and assessment of indoor pollutants in the residential houses of Hunan Tujia families and subsequently visualised and simulated fire pollutants in representative residential houses by using fire-dynamic-simulator software. Pollutant-control strategies, using passive smoke collectors and resizing windows, were proposed and simulated for validation. The results revealed that passive smoke collectors reduced the pollutant concentration in the hall house by 43.96%. Furthermore, the optimal window size was 1500 mm × 1500 mm, and the most reasonable windowsill height of the firepit was 1800 mm. The results of the study can be used to improve the indoor air quality of Tujia dwellings and mitigate the adverse health effects of exposure to indoor air pollution without restricting ethnic beliefs and traditional customs.

## 1. Introduction

People spend more than 80% of their lives indoors, and exposure to air pollution affects their health [1,2]. A minimum of 95% of the world’s population breathes air containing dangerously high levels of pollutants [3]. Furthermore, ambient air pollution leads to up to 4.2 million deaths annually [4]. Nearly 3 billion people still burn wood, animal manure, charcoal, and crop waste as the primary source of energy for cooking and heating, which leads to 3.8 million deaths from diseases caused by indoor air pollution [5,6,7]. In China, air pollution poses a serious health threat. Furthermore, in China, 1.2–1.6 million premature deaths are attributed to indoor air pollution [8,9]. Research on restricting indoor pollutant control remains challenging in China. In particular in rural China, approximately 46% of households use solid fuels (95% confidence interval (CI), 33–59%) [10]. Indoor solid-fuel utilization not only is a major cause of indoor air pollution but also increases the potential risks of many chronic diseases (e.g., heart disease, hypertension, and respiratory disease) and depression [10,11,12,13]. The traditional habits and unique ethnic fire culture of the Hunan Tujia region cause residents, especially the elderly, to be exposed to pollutants for a long term. Investigation and mitigation of the indoor pollutant situation are critical.

**Figure 1:**
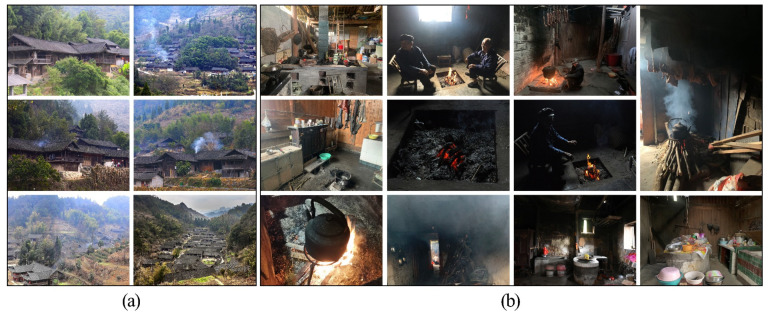
Hunan Tujia dwellings and their indoor pollutant conditions. (**a**) Hunan Tujia dwellings. (**b**) Indoor pollutant condition.

The Hunan Tujia region is a gathering place of traditional settlements in China. The Tujia are one of the ten most populous minority groups in China. The Tujia family preserves a unique fire culture with frequent traditional fire god rituals [14]. The firepit is not only the core area of life of the Tujia folk houses but also the place where families gather, receive guests, have discussions, and perform rituals [15]. Their ethnic beliefs and traditional culture centres around these firepits. In ancient times, the fire in the Tujia firepit was extinguished only once during the Spring Festival. The Tujia still retain their traditional fire-pond fire habits and culture. However, wood, charcoal, and coal are the most dominant firepit fuels, whereas solid fuels, such as coal and biomass are widely used as cooking and heating sources [16,17,18]. Thus, the living habits and ethnic culture of the Tujia people have resulted in long-term exposure to polluted environments (Figure 1). The Chinese government has introduced policies, such as subsidising clean-fuel technologies and encouraging clean fuels, to mitigate indoor air pollution [19]. However, their effects are unsatisfactory. Traditional fire habits and culture have been perpetuated for generations, and switching to other energy sources involves additional costs. Because of the accelerating rate of ageing in China, and the large number of young people leaving the villages to settle in the cities, senior citizens are the most prominent resident inhabitants of traditional Tujia dwellings. Therefore, investigating indoor pollutants in Hunan Tujia dwellings, studying the diffusion pattern of indoor pollutants, and developing scientific and reasonable pollutant control strategies are critical for reducing the adverse health effects of exposure to indoor air pollution, especially for senior citizens.

Indoor health issues and adverse health effects of exposure to air pollution have attracted considerable attention worldwide, especially since the COVID-19 outbreak [20,21]. Studies related to indoor air quality have primarily focused on objective field monitoring and assessment [22,23,24,25,26]. Seo et al. [27] investigated computer emissions of polycyclic aromatic hydrocarbons to assess the characteristics of indoor air pollution and potential levels of human exposure. Zhang et al. [28] assessed 30,139 participants from north-eastern China to investigate the relationship between chronic exposure to outdoor and indoor solid-fuel use and depressive symptoms. Zhu et al. [29] proposed measures to control indoor air pollution and discussed policy implications by studying indoor air pollution in a large integrated transportation hub in Shanghai. Dubey et al. [30] observed the efficiency of air purifiers (APs) in removing indoor air pollutants and evaluated the effectiveness of APs in reducing the concentration of various sizes of particulate matter (PM) and ions in indoor environments. Dionova et al. [31] proposed a fuzzy logic controller (FLC)-based EIAQ monitoring and control system by using the ambient-indoor-air-quality index (EIAQI) as the main reference indicator state level. Chen et al. [32] investigated the CO_2_ and PM_2.5_ concentrations in 86 air-conditioned classrooms in primary and secondary schools in southern Taiwan and synthesised the effects of fresh-air ventilation systems on classroom-indoor air quality. Xie et al. [33] investigated the indoor air quality of badminton courts during the wet season to investigate the indoor-air-quality characteristics of naturally ventilated public sports buildings. Zhu et al. [34] investigated the causes of discomfort in office buildings in various climatic zones in China by detailing pollutant concentration and health-risk-assessment data as well as information on human perception of indoor environment. He et al. [35] conducted surveys, urine collection, and spirometry on 70 rural college students before and after winter break and evaluated respiratory effects caused by coal stoves and kang during winter break in rural Gansu Province, China. Cai et al. [36] investigated real-time indoor CO_2_ and PM_2.5_ concentrations as well as air temperature, humidity, and air purification effectiveness in 33 classrooms in 21 schools in Beijing, China, to provide data for assessing indoor air quality in classrooms with air purification equipment. Furthermore, visualisation and quantitative analysis through numerical simulation of pollutant dispersion is the primary method of study. Tong et al. [37] investigated the effect of traffic-related air pollution on indoor air quality in naturally ventilated buildings through air-quality modelling with CFD.

According to relevant literature, most indoor-air-quality studies have been conducted near office buildings [38,39,40,41], schools [42,43,44,45], large public buildings [46,47,48,49], and shopping malls [50,51]. Limited studies have been conducted in indoor air quality in rural areas of Hunan, China. However, the living habits and ethnic culture of Hunan Tujia residents results in long-term exposure to pollutants, which is highly detrimental to their health. Therefore, conducting indoor air quality assessment of Hunan Tujia dwellings is critical. Therefore, we selected typical dwellings for indoor air (formaldehyde (HCHO), CO_2_, PM_2.5_, PM_10_) monitoring and conducted pollutant-dispersion simulations using PyroSim software for firepits (the most critical source of indoor pollutants) and proposed mitigation strategies to reduce the health risks of indoor pollutants in Tujia dwellings.

## 2. Methodology

### 2.1. Sampling Site

**Figure 2:**
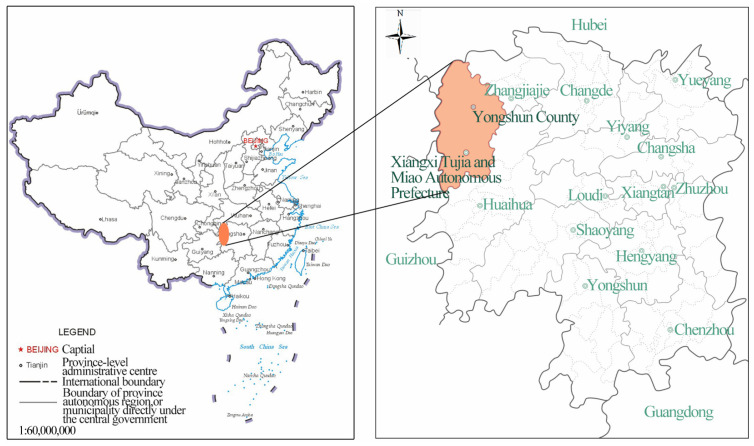
Location of Hunan Tujia in China and research location.

**Figure 3:**
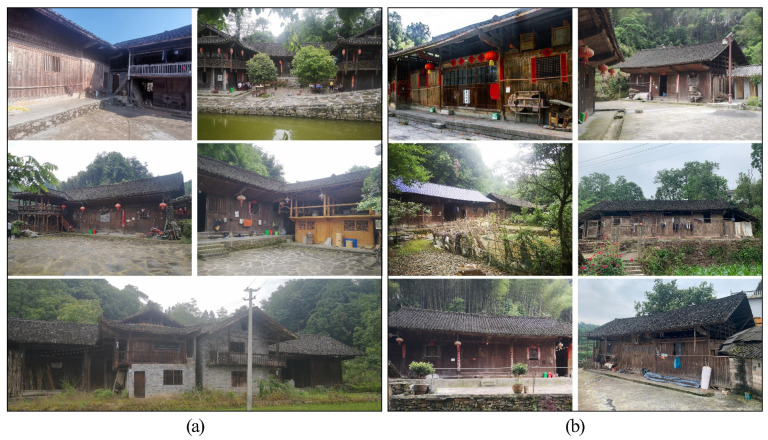
L-shaped and Zuozi dwellings in Hunan Tujia. (**a**) L-shaped dwellings. (**b**) Zuozi dwellings.

Hunan Tujia is mainly distributed in Yongshun, Longshan, Baojing, and Guzhang counties in Xiangxi Tujia and Miao Autonomous Prefecture; Cili and Sangzhi counties in Zhangjiajie City; and Shimen and other counties in Changde City (Figure 2). The unique regional environment, long history and culture, and traditional folk belief concepts of Tujia have resulted in their unique traditional architectural features and styles in western Hunan. We selected two representative dwelling types in Yongshun County, Hunan Province, namely the Zuozi dwelling and the L-shaped dwelling, and conducted indoor pollutant monitoring and simulation optimisation studies (Figure 3). The Zuozi dwelling is a single house with three openings in a “-” shape, whereas the L-shaped dwelling has an L-shaped layout with the main house and the rooms arranged at right angles because of the topographic constraints.

### 2.2. Questionnaire Survey

A questionnaire study on indoor air quality was conducted on Hunan Tujia residents. The questionnaire contained queries based on indoor air quality based on the study by Zhu et al. and additionally modified according to the characteristics of the Hunan Tujia residential environment [52,53,54]. The survey included living habits, awareness level of air pollutants, and subjective feelings regarding indoor air pollutants of the citizens (Table 1). A total of 900 questionnaires were distributed, and 764 valid questionnaires were finally returned.

### 2.3. Field Measurements

**Figure A1:**
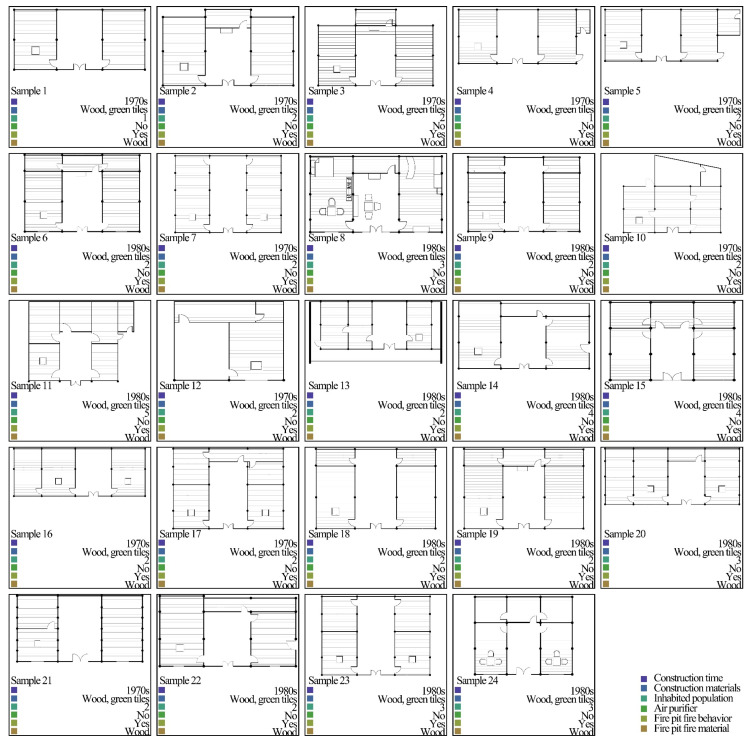
Information and living habits of the 23 Zuozi dwellings.

**Figure A2:**
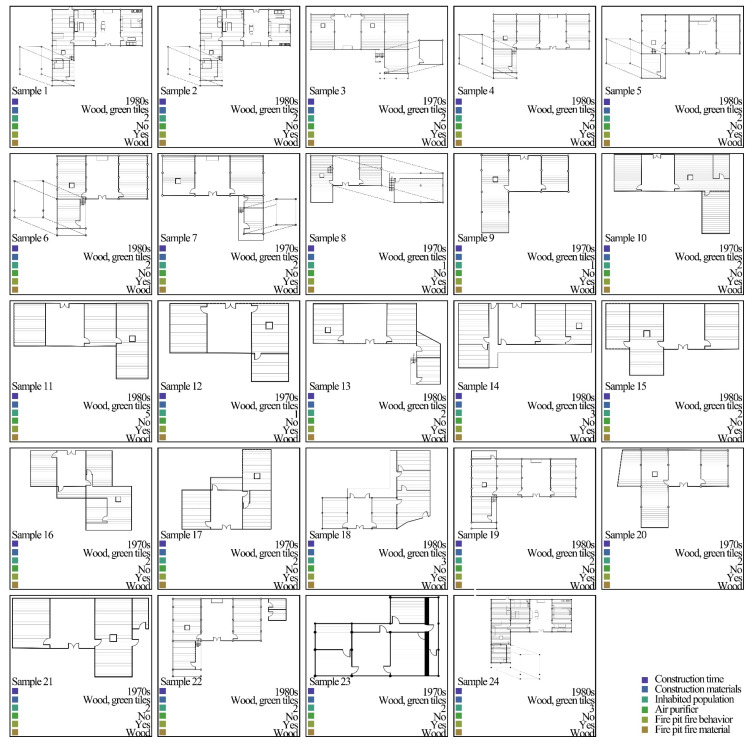
Information and living habits of the 23 L-shaped dwellings.

**Figure A3:**
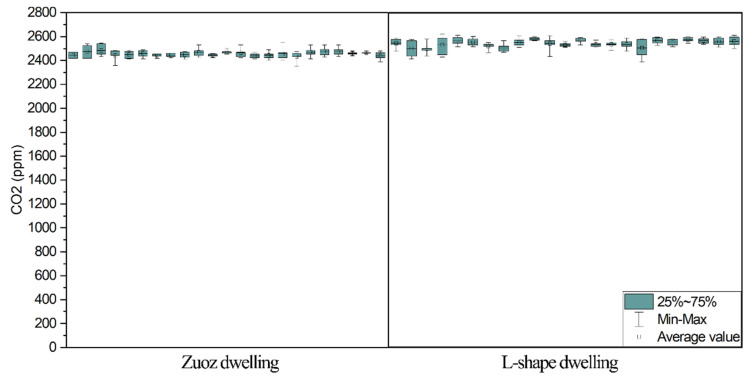
15-min CO_2_ concentration analysis in the firepits during the fire period of different Zuozi dwellings and L-shaped dwellings.

**Figure A4:**
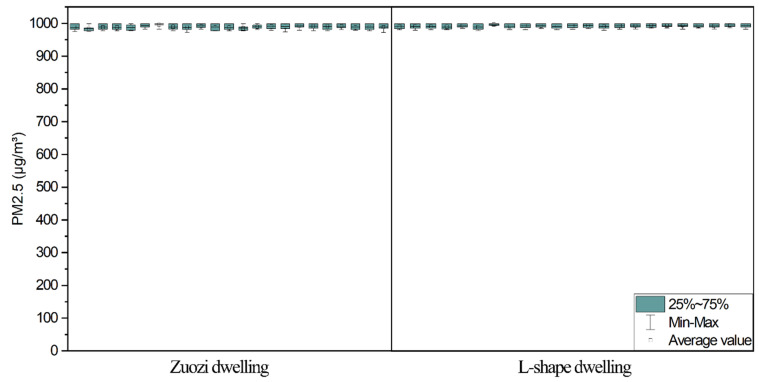
15-min PM_2.5_ concentration analysis in the firepits during the fire period of different Zuozi dwellings and L-shaped dwellings.

In the project, first, the approvals necessary for the study were obtained. After obtaining approval from the village chief and clerk, we conducted indoor air research and monitoring studies on dwellings. We conducted extensive mapping of Hunan Tujia dwellings, including 23 Zuozi dwelling dwellings and 23 L-shaped dwellings. The results of the survey showed that the Tujia dwellings had the same building materials, building structures, types of electricity, and fire habits (Figure A1 and Figure A2). Meanwhile, the traditional Tujia villages were located in the tree-rich mountainous area without any factories of pollutants. In addition, due to economic development, many young people went to cities to work and live, and the resident population here was mainly the elderly and children. Before the sample selection, we monitored the CO_2_ and PM_2.5_ concentration in the firepit rooms of 23 Zuozi dwellings and 23 L-shaped dwellings for 15 min during the fire-use period, and the results showed that the CO_2_ and PM_2.5_ concentration in the firepit rooms of different Zuozi dwellings was not very different (Figure A3 and Figure A4). The dwellings tested were selected by conductiong assessment in the field and discussion with the village chief and residents to ensure that these dwellings were representative but not disruptive to the normal life of the residents. The Zuozi dwelling was occupied by an elderly couple. The L-shaped dwelling was occupied by an elderly couple and one of their grandchildren, that is, three people. The selected dwellings were typical Tuozi dwellings, and their construction materials mainly included wood and small green tiles. The date 26 December 2021, was selected for indoor air quality monitoring. Before monitoring, the selected residences were subjected to daily sanitary cleaning. During the monitoring process, the residents performed their daily living activities, and the windows on the south side of all rooms and the doors of the hall were open.

**Figure 4:**
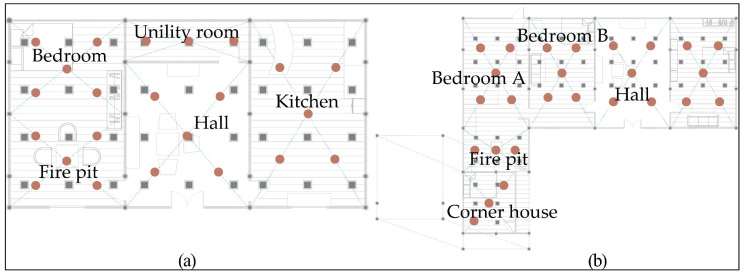
Pollutant monitoring points. (**a**) Zuozi dwelling monitoring points. (**b**) L-shaped dwelling monitoring points.

The two representative dwellings of the Tujia were selected for on-site measurements, including CO_2_ concentration, PM_2.5_ concentration, PM_10_ concentration, and HCHO concentration, by using AZ-77597 CO_2_ analyser and BR-SMART128S air-quality instruments. According to the Indoor Air Quality Standard [55,56], five monitoring points were set up in rooms of 50–100 m^2^ area, including the hall and bedroom, and three monitoring points were set up in rooms with area less than 50 m^2^, which included bedroom B, bathroom, kitchen, and utility room. The sampling points in this study were evenly distributed on the diagonal or pentagonal of each room. The instrument was placed at the measurement site approximately 1.1 m high from the ground, and the pollutant concentration was recorded at an interval of 3 min. The average value of each monitoring point is considered as the pollutant concentration of the room. The monitoring rooms of the Zuozi dwelling include firepits, bedrooms, parlours, and kitchens. The monitoring rooms of the L-shaped dwelling include corner houses, bedroom As, parlours, and bedroom Bs. The measurement time includes the time when the firepit is not in use for a long time (0–10 min), when it is in use, and when smoke is released (10–40 min). Furthermore, the case when the firepit is not in use and no smoke is released (40–95 min) is also considered. The monitoring instrument parameters are presented in Table 2. The monitoring points of each room are displayed in Figure 4.

### 2.4. Data Analysis

Based on the residential building design code (GB50340-2016) [57,58] (Table 3), the indoor pollutant concentration was employed to evaluate and analyse the effect of the PM concentration on indoor air quality and human respiration. Moreover, the indoor concentration of various pollutants under the stable working condition of fire with firepits was evaluated. The Indoor Air Quality Standard assignment is presented in Table 4.

However, residents exposed to high levels of formaldehyde for long periods of time are at risk of developing cancer. According to the United States Environmental Protection Agency health-risk-assessment model [60,61], and with reference to the Ministry of Environmental Protection and the China Environmental Exposure Behavior Study, the health risk of formaldehyde can be calculated by using the following equation.

(1) CR=I×CSF

where CR is the carcinogenic risk, I is the daily intake, mg·(kg·d)^−1^, CSF indicates the carcinogenic slope factor kg·d·mg^−1^, and formaldehyde is 0.046 kg·d·mg^−1^.

### 2.5. Indoor-Pollutant-Spreading Software Simulations

In residential dwellings, firepits were the source of indoor pollutant generation. We proposed a targeted indoor-pollutant-concentration control strategy in which the process of smoke release from an indoor firepit in the traditional Zuozi dwelling was simulated using PyroSim simulation [62,63] software to visualize the dispersion of pollutants released from the firepit for numerical study.

#### 2.5.1. Simulation Methods

**Figure 5:**
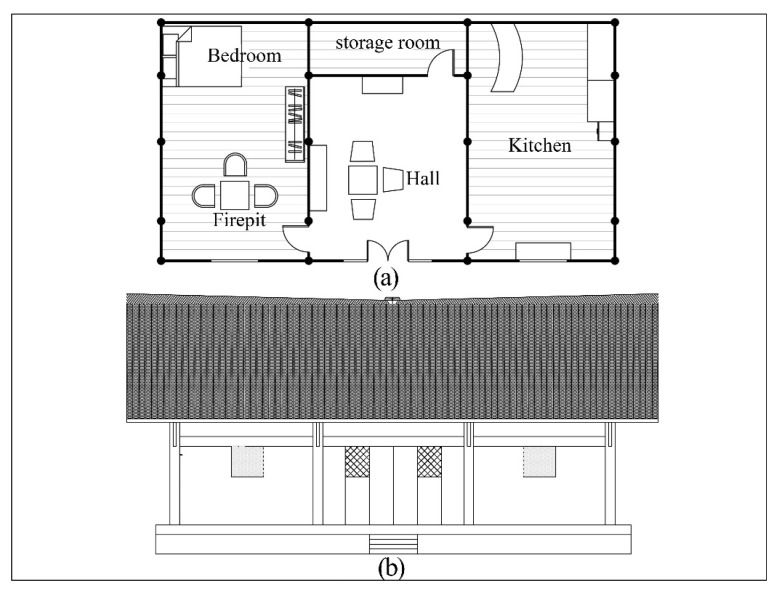
Building plan and elevation. (**a**) Layout plan. (**b**) South elevation.

A typical Zuozi dwelling of a Tujia family was selected for indoor-pollutant-dispersion simulation with fire-dynamics-simulator (FDS) software. The building plan is in the form of three large rooms, three small rooms, and five columns, with an opening of 12 m, a depth of 6.5 m, and a height from the ground to the eaves of 6.5 m. The form and volume of this house are representative of the western Hunan region. The building has one floor with a bedroom, firepit, hall, kitchen, and utility room (Figure 5).

#### 2.5.2. Model Building and Parameter Selection

##### Simulation Model

**Figure 6:**
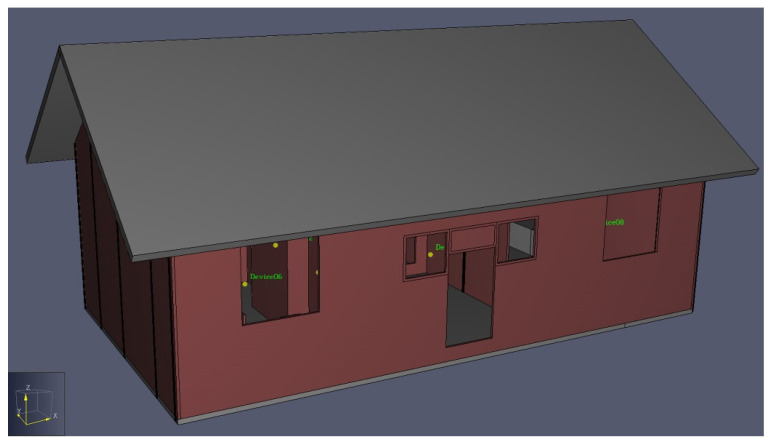
Simulation model.

The model of the simulation was developed using PyroSim software based on field mapping data, and the indoor furniture of the residential house was arranged according to a real-world situation. The firepit is the source of indoor pollutants, and the fire source size was 0.4 m × 0.4 m, according to the true size and location of the firepit. Considering the living habits of the residents, all doors and windows of the building were to be opened (Figure 6).

##### Parameter Setting

Because the simulation process is low-Mach, large eddy simulation was selected in the paper [64]. The Smagorinsky coefficient *C*_s_, turbulence Prandtl number *P*_rt_, and turbulence Schmidt number *S*_ct_ were 0.2, 0.5, and 0.5, respectively [65]. Based on the simulated residential situation, the dimensions of the simulated calculation area were set to 14 m × 8.5 m × 6.5 m, and the volume of the calculation area was 773.5 m^3^. According to the user manual of PyroSim software, the grid size was set to 0.25 m × 0.25 m × 0.25 m, with a total of 49,504 grids. Combined with the maximum heat release rate of the fire source of the firepit, the grid setting was verified based on the equation of the grid division method in the PyroSim software user manual. The ratio of the fire source feature diameter to the grid size was 7.692, and the simulation result was consistent with the grid independence test result.

(2) $$D^{*}={\left(\frac{Q}{\rho_{0}c_{p}T_{0}\sqrt{g}}\right)}^{\frac{2}{5}}$$

where $D^{*}$ is the fire source feature diameter, Q is the heat release rate of the fire source, taken as 2000 kW; $\rho_{0}$ is the air density, taken as 1.205 kg/m^3^; $c_{p}$ is the specific heat capacity of air, taken as 1.004 kJ/(kg k); $T_{0}$ is the ambient temperature, taken as the annual average temperature of the Tujia region (i.e., 293.15 k); and *g* is the acceleration of gravity, taken as 9.8 m/s^2^ [66,67].

(3) $$\delta_{x}=\sqrt[{3}]{x\times y\times z}$$

where $\delta_{x}$ is the grid size, and *x*, *y*, and *z* are the dimensions of the X, Y, and Z coordinate axes of the unit grid, respectively.

**Figure 7:**
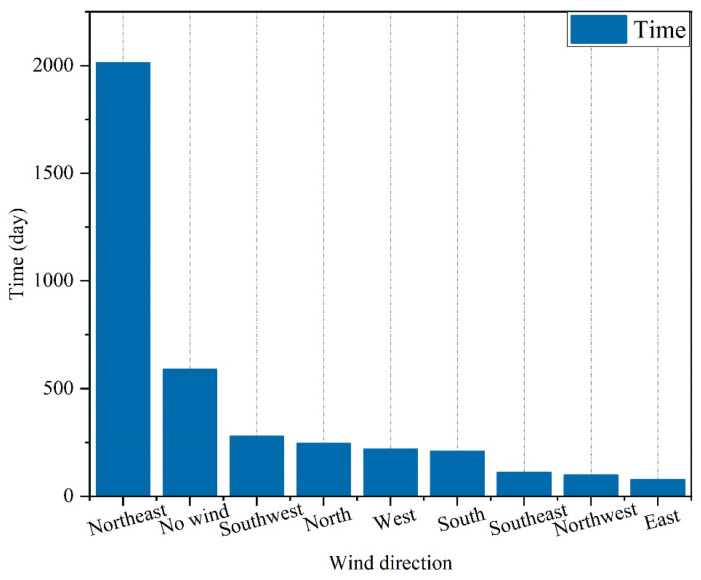
Historical wind statistics of Hunan region from 2011 to 2022.

From the statistics of China Meteorological Administration, we obtained the historical wind direction in the Hunan region from 2011 to 2022, in which the north-easterly wind accounts for 52.07% of the time (Figure 7). Therefore, the wind speed in the simulation was set to 1.5 m/s from the northeast. The simulation time was set to 1000 s.

### 2.6. Pollutant Control Strategies

#### 2.6.1. Passive Smoke Collector

**Figure 8:**
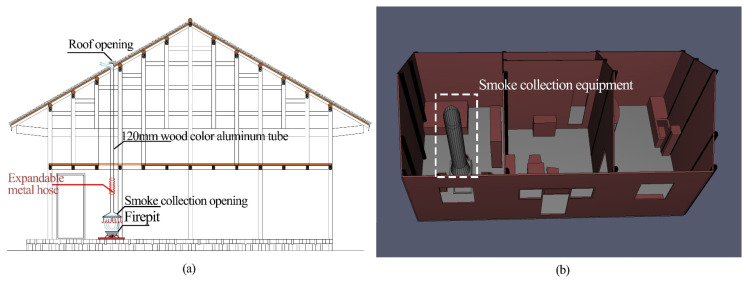
Passive smoke collector and its simulation model. (**a**) Passive smoke collection equipment. (**b**) Passive smoke collection equipment simulation model.

A special skylight-cat window (i.e., a small hole of 300 mm × 300 mm) was designed on the roof of Hunan Tujia Zuozi dwellings (Figure 8a). We installed a passive smoke collector above the firepit (Figure 8b). The principle of the thermal pressure difference was used to make a large amount of smoke, which was rapidly exhausted outside through the collector pipe, passing through the cat window. The selected passive smoke collectors were universal and not specific. Thus, the indoor air quality was improved. To verify the effectiveness of the passive smoke collector, we simulated the indoor-pollutant-concentration dispersion with this device installed. The simulation model is displayed in Figure 1. The parameters of the simulation were set the same as in Section 2.5.2.

#### 2.6.2. Window Size

**Figure 9:**
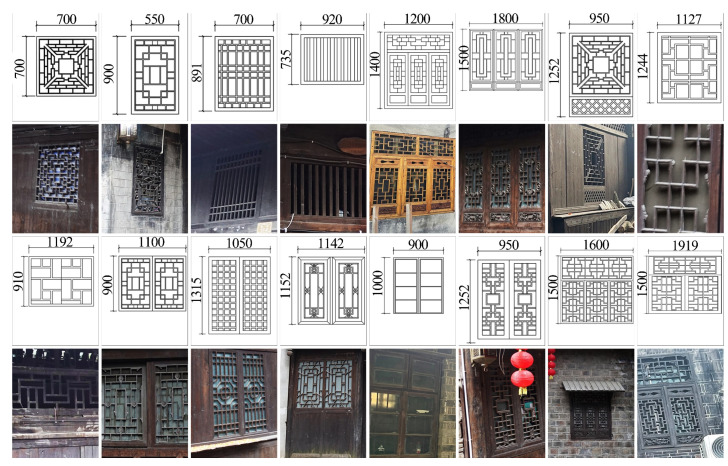
Window size of traditional Hunan Tujia dwellings.

Window size directly affects indoor ventilation. The appropriate window size can help reduce the indoor pollutant concentration. The use of solid fuels in firepits is the most crucial source of indoor pollutants in Tujia dwellings. The indoor air quality in the firepit room was the poorest when the firepit was closer to the bedroom than the kitchen. Therefore, this study was conducted in the firepit room. The windows of traditional Hunan Tujia dwellings were 700–1500 mm long and 550–1800 mm wide (Figure 9). We simulated indoor pollutant dispersion under the working conditions of different window sizes of 900 mm × 900 mm, 1200 mm × 1200 mm, 1350 mm × 1350 mm, 1400 mm × 1400 mm, 1450 mm × 1450 mm, 1500 mm × 1500 mm, 1800 mm × 1800 mm, and 2100 mm × 2100 mm. The parameters of the simulation were the same as those presented in Section 2.5.2.

#### 2.6.3. Windowsill Height

The windowsill height is another factor that affects indoor ventilation. An appropriate windowsill height helps pollutants diffuse to the outdoors. In Tujia dwellings, 900 mm is the most common windowsill height. To obtain more light and ventilation, many dwellings also have secondary windows, which are additional windows on the top of windows and doors. The height of the secondary windows is 2400–2700 mm. To determine the appropriate windowsill height for Zuozi dwellings, we simulated indoor pollutant concentration dispersion under different working conditions of the windowsill height of firepit rooms, where the windowsill heights were 900, 1200, 1500, 1800, 2100, 2400, and 2700 mm. For the size setting of the windowsill, we selected the height of 900–2700 mm for the study, considering the various types of windows in Tujia dwellings. This range is feasible in real life. The simulation parameters are the same as those presented in Section 2.5.2.

## 3. Results

### 3.1. Questionnaire Survey Result

**Figure 10:**
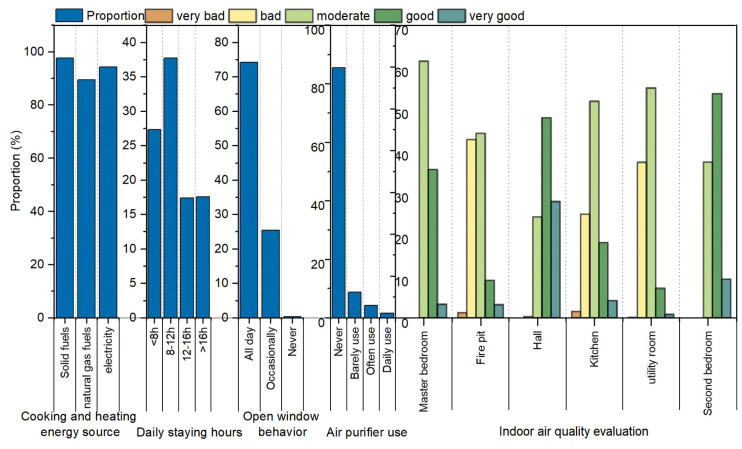
Questionnaire survey result.

The results of the questionnaire survey are displayed in Figure 10. The results revealed that 34.17% of people who participated in the survey were over 60 years old and 71.4% were women. Hunan Tujia is an ageing population; therefore, indoor air quality in the homes of the elderly persons should be studied. Solid fuels (such as wood, charcoal, and coal) are the main source of energy for cooking and heating. The use of solid fuels increases the concentration of indoor pollutants and is one of the important sources of pollutants in Tujia dwellings. Residents typically open windows, with 74.28% of people opening windows throughout the day. However, the use of APs is limited, and the percentage of people who never use APs is more than 80%. The percentage of people staying indoors for 8–16 h daily is 55.1%. A highly polluted environment poses a severe threat to the health of residents. The firepit and kitchen had the worst indoor air quality assessment environment and were rated as moderate with 44.13% and 51.79%, respectively. Residents in the pollutant environment become acclimatised to an environment. Because of the degradation of organ functions of some elderly people, these people are not sensitive to the degree of air pollutant. Therefore, Hunan Tujia residential indoor pollutants should be controlled based on senior care.

### 3.2. Field Measurements Result

#### 3.2.1. HCHO

**Figure 11:**
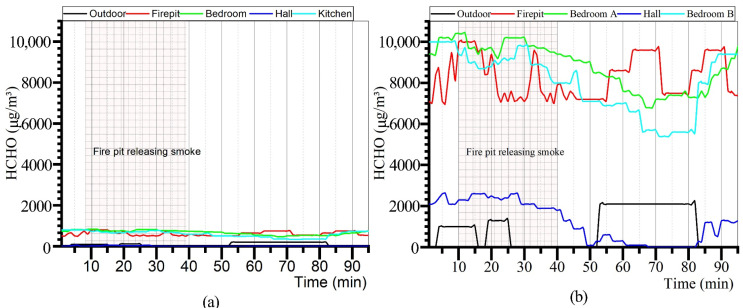
Measurement of the indoor HCHO concentration in Zuozi and L-shaped dwellings. (**a**) Indoor HCHO measurement results of Zuozi dwelling. (**b**) Indoor HCHO measurement results of L-shaped dwelling.

The results of the indoor HCHO concentration monitoring in Zuozi and L-shaped dwellings are displayed in Figure 11. The results revealed that the indoor HCHO concentrations in the Zuozi dwelling and the L-shaped dwelling vary irregularly and are not affected by the firepit. The hall of western Hunan dwellings is a place used for meeting, chatting, and entertainment, with the largest door. When people are in the residence, the hall door is always open; thus, the hall space has the optimum ventilation among all the rooms. With excellent ventilation, the hall of both the Zuozi dwelling and the L-shaped dwelling is the room with the lowest indoor HCHO concentration. The indoor HCHO environment of the Zuozi dwelling is considerably better than that of the L-shaped dwelling. The selected L-shaped and Zuozi dwellings have the same building materials and are located in the same environment, and the residents have the same living habits. The difference in indoor concentrations between the Zuozi dwelling and the L-shaped dwelling was caused by the interior furniture and decoration materials. Furthermore, no significant change in HCHO was observed in all rooms of both the Zuozi dwelling and the L-shaped dwelling during the fire period. This result indicated that the firepit fire behavior does not cause a considerable increase in indoor HCHO concentrations.

**Figure 12:**
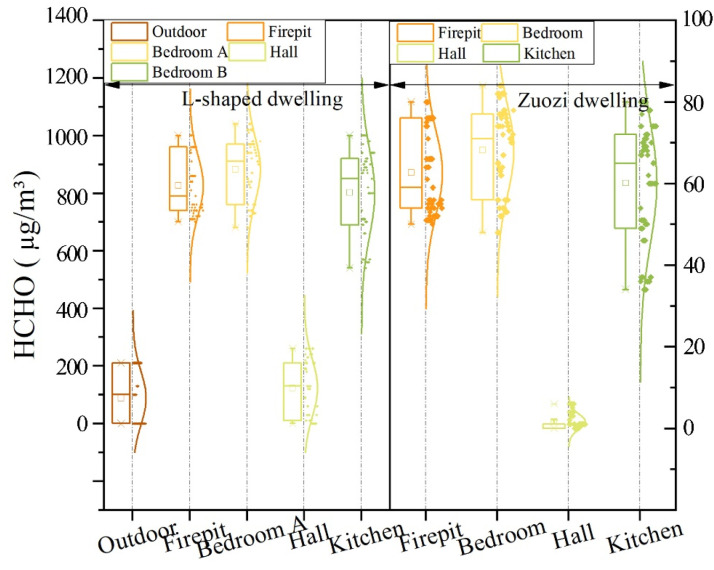
Comparison of the indoor HCHO concentration between Zuozi and L-shaped dwellings.

The analysis results of HCHO concentrations for each room of the Zuozi dwelling and the L-shaped dwelling are displayed in Figure 12. The mean indoor HCHO concentration (658 μg/m^3^) in the L-shaped dwellings was considerably higher than the Chinese standard. The indoor carcinogenic risk of formaldehyde in the Zuozi dwellings was 5.748 × 10^−5^ for men and 5.382 × 10^−5^ for women, and the indoor carcinogenic risk of formaldehyde in the L-shaped dwelling was 7.88 × 10^−4^ for men and 7.38 × 10^−4^ for women. For Hunan Tujia dwelling, the carcinogenic risk values caused by formaldehyde exceeded the range of formaldehyde recommended by the U.S. Environmental Protection Agency [68]. In western Hunan, the indoor HCHO concentrations in traditional dwellings without new decoration were in accordance with the Chinese regulations. However, the indoor HCHO concentrations of newly renovated dwellings were higher than the Chinese regulation values. The residents living in these dwellings are highly prone to cancer with high HCHO concentrations for a long time. Therefore, we suggest that for newly decorated residential houses, more attention should be paid to indoor HCHO concentrations to avoid serious health risks.

#### 3.2.2. CO_2_

**Figure 13:**
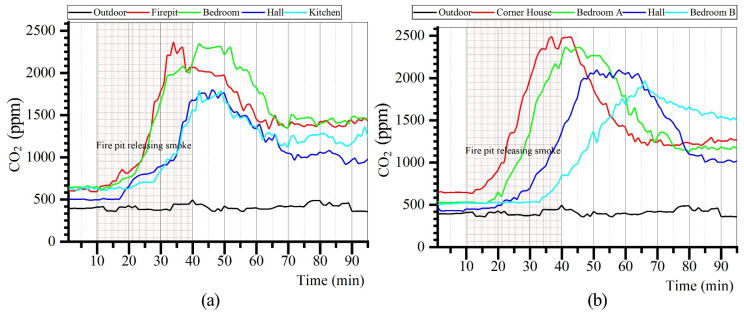
Measurements of the indoor CO_2_ concentration in Zuozi and L-shaped dwellings. (**a**) Indoor CO_2_ measurement results of Zuozi dwelling. (**b**) Indoor CO_2_ measurement results of L-shaped dwelling.

The measured results of CO_2_ concentrations in each room of the Zuozi dwelling and L-shaped dwelling are displayed in Figure 13. The CO_2_ concentration in each room of the Zuozi dwelling and L-shaped dwelling increased rapidly during the time when the fire was used by the indoor residents. After the residents stopped using fire, the indoor CO_2_ concentration peaked in the following period and subsequently gradually decreased. Among them, the firepit was the room with the fastest increase in CO_2_ concentration. During the air-monitoring period, the CO_2_ concentrations in all indoor rooms were higher than those outdoors. The trend of the CO_2_ concentration change in each room indoors remained the same for Zuozi and L-shaped dwellings. The CO_2_ concentrations in the firepit and bedroom of the Zuozi dwelling were always higher than those in the hall and kitchen, and the CO_2_ concentration in the hall was the least variable, whereas the concentration in the L-shaped house varied in the order of the corner house, bedroom A, the hall, and bedroom B. The kitchen of the Zuozi dwelling was the farthest from the firepit, the hall was the room with the optimum indoor ventilation, and the bedroom was the closest to the firepit; therefore, the CO_2_ concentration in the firepit and bedroom of the Zuozi dwelling was always higher than that in the hall and kitchen, and that in the hall varied the least. In addition, bedroom B and the corner room were farthest and closest from the firepit, respectively. The hall of the L-shaped dwelling is the room with the otpimum indoor ventilation. Therefore, the order of concentration changes in the L-shaped dwelling is the corner room, bedroom A, the hall, and bedroom B.

**Figure 14:**
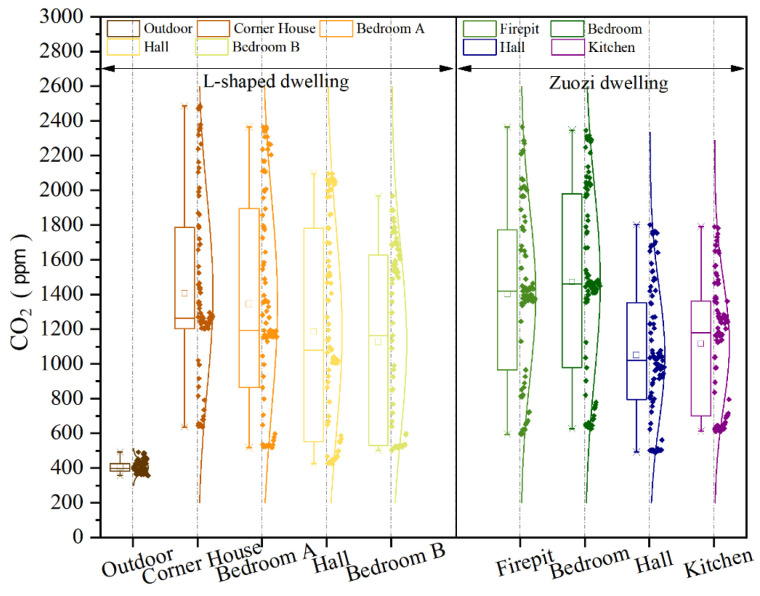
Comparison of indoor CO_2_ concentration between Zuozi and L-shaped dwellings.

The analysis results of CO_2_ concentrations in each room of Zuozi and L-shaped dwellings are displayed in Figure 14. The mean values of the CO_2_ concentration in the corner house, bedroom A, hall, and bedroom B of the L-shaped dwelling were 1407, 1345, 1185, and 1126 ppm, respectively. The highest indoor CO_2_ concentration was in the corner house of the L-shaped dwelling (2486 ppm). The CO_2_ concentration in the stable state of the indoor air in the L-shaped dwelling’s mill foot house, bedroom, hall house, and bedroom were 1274, 1156, 1019, and 1497 ppm, respectively, which corresponded to the evaluation assignment of 3, 3, 3, and 3, and the degree of air quality was average. The average values of CO_2_ concentrations in the firepit room, bedroom, parlour, and kitchen of the Zuozi dwelling were 1403, 1471, 1051, and 1116 ppm, respectively, and the CO_2_ concentrations in the bedroom and firepit varied the most, with a range of over 1700 ppm. The CO_2_ concentration in the steady state of indoor air in the Zuozi dwelling, the hall, and the kitchen were 1530, 1550, 981, and 1241 ppm, respectively, and the evaluation assignment values were 2, 2, 3, and 3, and the degree of air quality was poor.

#### 3.2.3. PM_2.5_

**Figure 15:**
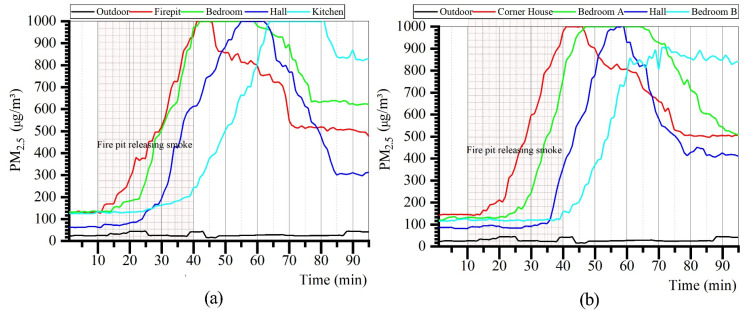
Actual measurement of indoor PM_2.5_ concentration in Zuozi and L-shaped dwellings. (**a**) Indoor PM_2.5_ measurement results of Zuozi dwelling. (**b**) Indoor PM_2.5_ measurement results of L-shaped dwelling.

The measured results of PM_2.5_ concentrations in each room of Zuozi and L-shaped dwellings are displayed in Figure 15. The trend of indoor PM_2.5_ concentration in each room was consistent with that of CO_2_, and the PM_2.5_ concentration is influenced by indoor fire behaviour. During the time when the indoor residents used fire, the indoor CO_2_ concentrations in each room of the seated house and L-shaped dwelling increased rapidly. After the residents stopped using fire, the indoor CO_2_ concentration peaked in the following period and subsequently gradually decreased. Among them, the room with the firepit exhibited the fastest increase in PM_2.5_ concentration. During the air-monitoring period, the PM_2.5_ concentrations in each room indoors were higher than those outdoors. The order of PM_2.5_ concentration changes in the Zuozi dwelling was firepit, bedroom, hall, and kitchen, whereas the order of PM_2.5_ concentration changes in the L-shaped house was corner room, bedroom A, hall, and bedroom B.

**Figure 16:**
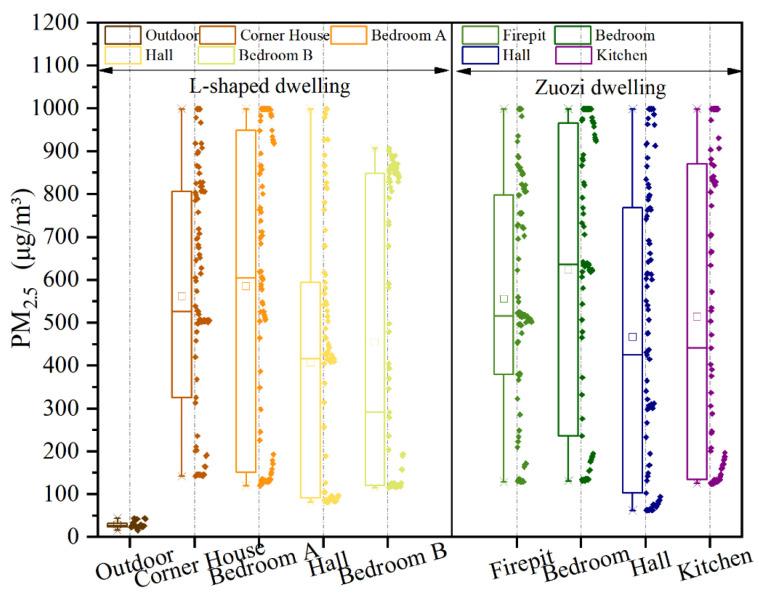
Comparison of the indoor PM_2.5_ concentration between Zuozi dwelling and L-shaped dwelling.

The analysis results of PM_2.5_ concentrations in each room of the Zuozi dwelling and the L-shaped dwelling are displayed in Figure 16. The mean PM_2.5_ concentrations in the corner house, bedroom A, hall, and bedroom B of the L-shaped dwelling were 562, 586, 407, and 455 μg/m^3^, respectively. The variation in PM_2.5_ concentrations in each room was not significant. The maximum indoor PM_2.5_ concentration also exhibited limited variation (984 μg/m^3^). The steady-state indoor air PM_2.5_ concentrations in the L-shaped residential house were 498, 495, 410, and 842 μg/m^3^ in the corner house, bedroom A, the hall, and bedroom B, respectively, and their evaluation correction values were 2, 2, 2, and 1, respectively. The air-quality degree was average. The mean PM_2.5_ concentrations in the firepit room, bedroom, hall, and kitchen of the Zuozi dwelling were 556, 624, 467, and 515 μg/m^3^, respectively, and the variation of PM_2.5_ concentrations in each room was not significant. The steady-state indoor air PM_2.5_ concentrations in the firepit, bedroom, hall, and kitchen of the Zuozi dwelling were 476, 622, 310, and 829 μg/m^3^, respectively, and the evaluation assignment values were 2, 1, 2, and 1, and the degree of air quality was poor.

#### 3.2.4. PM_10_

**Figure 17:**
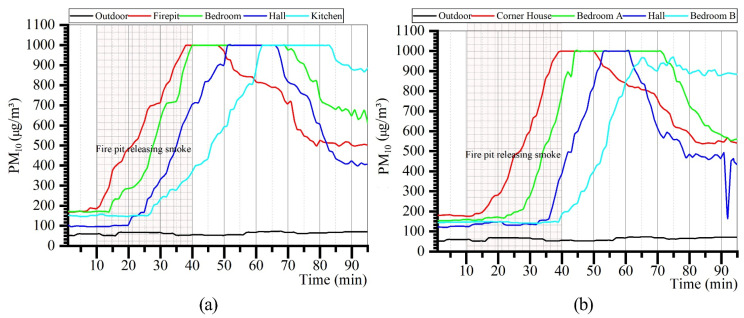
Actual measurement of the indoor PM_10_ concentration in Zuozi and L-shaped dwellings. (**a**) Indoor PM_10_ measurement results of Zuozi dwelling. (**b**) Indoor PM_10_ measurement results of L-shaped dwelling.

The measured results of PM_10_ concentrations in each room of the Zuozi and the L-shaped dwelling are displayed in Figure 17. The trend of the indoor PM_2.5_ concentration change in each room is consistent with that of CO_2_ and PM_2.5_. The PM_10_ concentration is affected with the indoor fire-use behaviour. The change in the PM10 concentration in each room increased rapidly when fire was used indoors, and subsequently reached the peak. The rooms most affected by indoor fire-use habits were the firepit and the corner in the Zuozi and L-shaped dwellings, respectively. The indoor PM_10_ concentrations in each room were higher than those outdoors during the indoor air-monitoring period.

**Figure 18:**
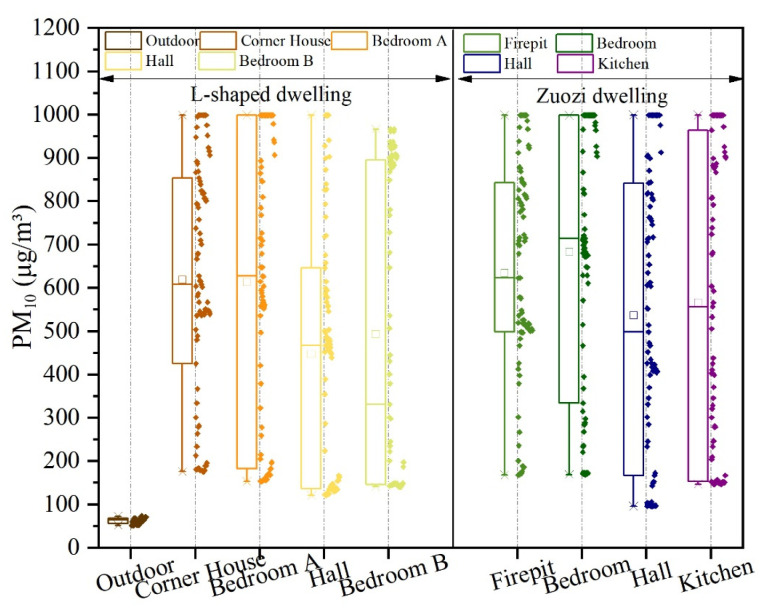
Comparison of the indoor PM_10_ concentration between Zuozi and L-shaped dwellings.

The analysis results of PM_10_ concentrations in each room of the Zuozi and the L-shaped dwelling are displayed in Figure 18. The mean PM_10_ concentrations in the corner house, bedroom A, hall, and bedroom B of the L-shaped dwelling were 619, 613, 447, and 494 μg/m^3^, respectively. The maximum indoor PM_10_ concentration was not different (992 μg/m^3^). The steady-state indoor air concentrations in the L-shaped dwelling were 498, 495, 410, and 842 μg/m^3^, and their evaluation values were 2, 2, 2, and 1 for the corner room, bedroom A, the hall, and bedroom B, respectively. The overall air-quality level was normal. The mean PM_10_ concentrations in the firepit, bedroom, hall, and kitchen of the Zuozi dwelling were 635, 683, 537, and 566 μg/m^3^, respectively, and the variation of PM_2.5_ concentration in each room was not significant. The steady-state indoor air PM_10_ concentrations in the firepit room, bedroom, hall, and kitchen of the Zuozi dwelling were 502, 595, 407, and 887 μg/m^3^, respectively, with evaluation assignments of 2, 2, 3, and 1. The overall air-quality degree was normal.

### 3.3. Software Simulation Results

**Figure 19:**
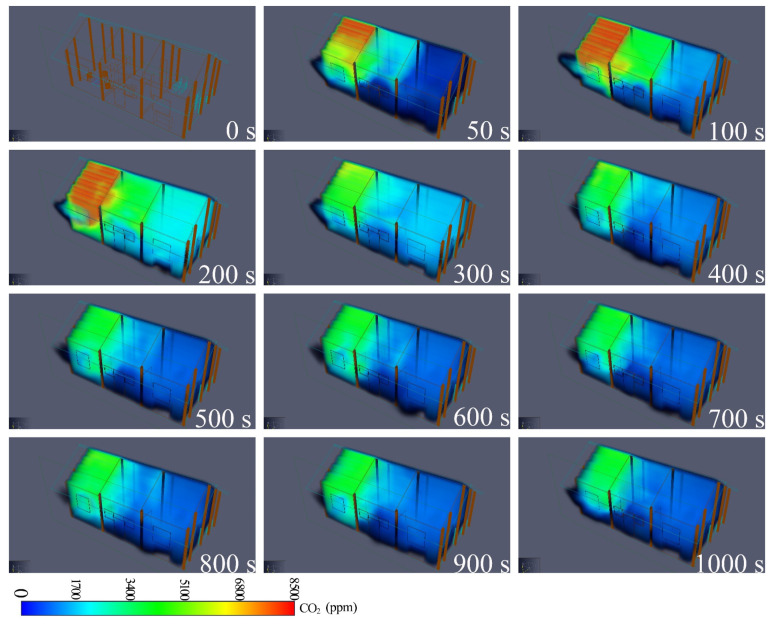
Simulation results of the CO_2_ release process from the firepit.

**Figure A5:**
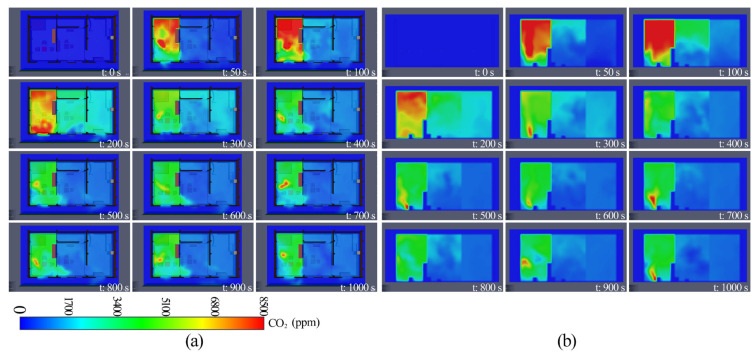
Indoor CO_2_ dispersion over time in each room. (**a**) Spread of indoor pollutants in the horizontal direction. (**b**) Spread of indoor pollutants in the vertical direction.

**Figure A6:**
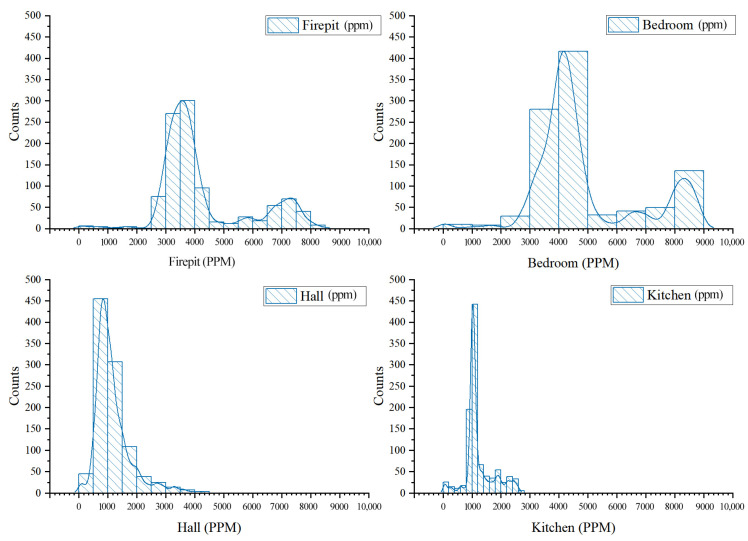
Indoor CO_2_ concentration in each room over time.

The simulation results of the smoke release process from the firepit are displayed in Figure 19. The indoor dispersion of CO_2_ over time in each room is shown in Figure A5 and Figure A6. The process of indoor pollutant diffusion caused by firepits with fire can be categorised into three stages. The first stage is the rapid spread at the fire-source site. Within 50 s after the ignition of the fuel in the firepit, the smoke spreads rapidly from the firepit to the bedroom and fills the entire firepit and the headspace of the bedroom. In the second stage, fire spread from the fire source site to other rooms. After 100 s, the smoke spreads to the hall and at 200 s, fire spreads to the kitchen. The smoke concentration in the kitchen is low because all windows, and the door to the hall are open and smoke spreads to the outdoors through the windows and doors. In the third stage, smoke dissipates to the outdoors. In addition, when the solid fuel in the firepit burned out and no combustible material was available in the annex, the smoke concentration in the firepit did not increase after 200 s. However, indoor smoke concentrations remained considerably higher in the firepit and bedroom closest to the firepit than in the other rooms after 200 s due to the increase in indoor pollutants caused by the fire behavior in the firepit. With the cessation of fire behaviour in the firepit, smoke concentrations decrease in all rooms indoors. Considering the living habits of residents with open windows, the doors and windows of all the rooms and the doors of the parlor were kept open during the simulation period. The CO_2_ concentrations were considerably lower in the window and door areas of the parlor and bedroom than in other areas.

## 4. Pollutant Control Strategies

### 4.1. Passive Smoke Collector

**Figure 20:**
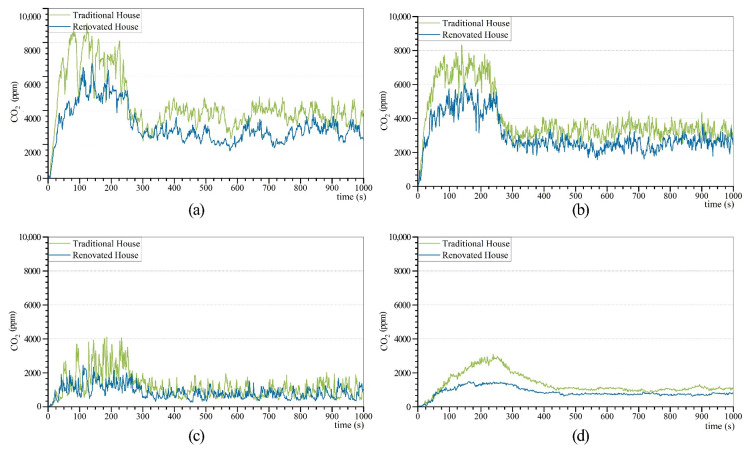
Simulation results of the CO_2_ concentration in each room in rooms with or without passive smoke collector. (**a**) Bedroom pollutants of installed equipment over time. (**b**) Firepit pollutants of installed equipment over time. (**c**) Hall pollutants of installed equipment over time. (**d**) Kitchen pollutants of installed equipment over time.

**Figure A7:**
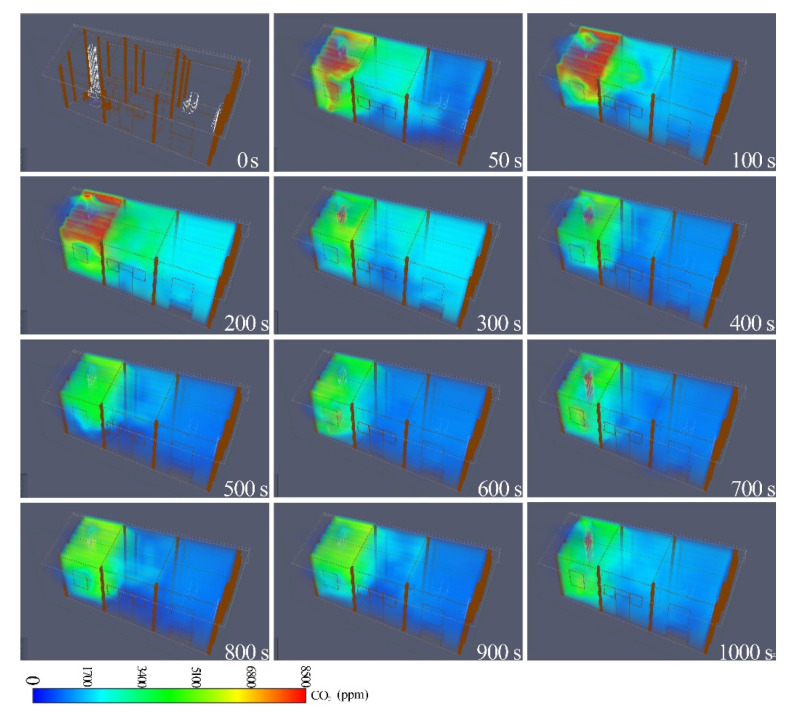
Simulation results of the CO_2_ release process of dwelling with passive smoke collector installation.

The results of the CO_2_ concentration over time for each room in rooms with or without passive smoke collector installation are displayed in Figure 20. The simulation results of the smoke release of dwellings with passive smoke collectors are displayed in Figure A7. During the simulation time, the CO_2_ concentrations in all rooms with passive smoke collectors installed were lower than those in the traditional residential house. The peaks of the curves of the modified bedroom, firepit, hall, and kitchen were 34.78%, 36.29%, 67.16%, and 108.5% lower than the conventional peaks, respectively.

**Figure 21:**
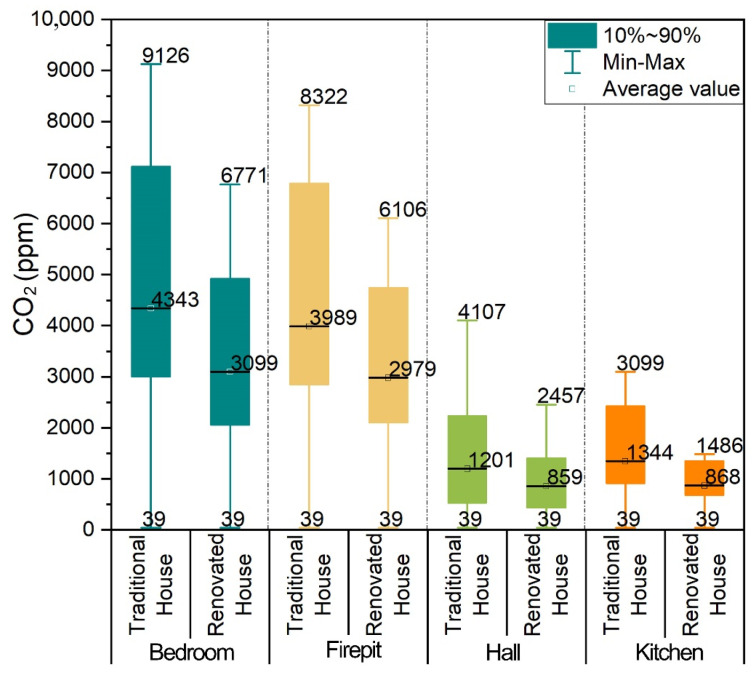
Comparison of CO_2_ concentration in each room in rooms with or without passive smoke collector.

The statistical comparison of CO_2_ concentration in each room in rooms with or without passive smoke collector is displayed in Figure 21. The maximum and average values of the CO_2_ concentration in each room of the renovated house were lower than those of the conventional one. The average values of the CO_2_ concentration in the modified bedroom, firepit, hall, and kitchen were 43.96%, 43.52%, 83%, and 118.12% lower than the conventional values, respectively. The installation of passive smoke collectors is an effective method to improve the indoor air quality in Zuozi dwellings.

### 4.2. Window Size

**Figure 22:**
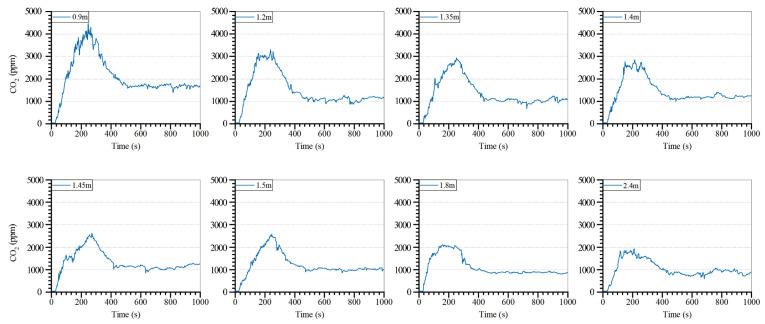
CO_2_ concentration in firepit room with different window sizes.

The results of the CO_2_ concentration in the firepit for different window sizes are displayed in Figure 22. The trend of the indoor CO_2_ concentration with time is the same for different window sizes. In all window sizes, the indoor CO_2_ concentration rapidly increased to the peak and subsequently decreased. With the increase in the window size, the curve of the CO_2_ concentration over time became flat. The highest indoor CO_2_ concentrations under the eight working conditions are distributed as 4470, 3300, 2930, 2850, 2620, 2570, 2110, and 1940 ppm.

**Figure 23:**
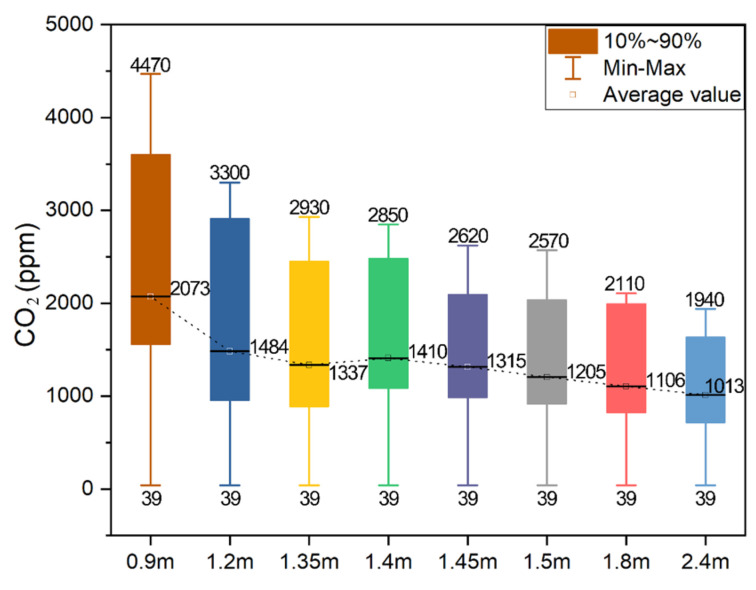
Analysis of the CO_2_ concentration in the firepit with various window sizes.

The result analysis of the CO_2_ concentration in the firepit with different window sizes is displayed in Figure 23. The larger the windows size, the smaller the maximum and average indoor CO_2_ concentrations, and the lesser the pollutants caused by fire habits in the firepit room. However, with the increase in the window size, the magnitude of the CO_2_ concentration change decreased, and the indoor pollutant control effect was less obvious. The average indoor CO_2_ concentration of 1400 mm × 1400 mm window size was 56.84% lower than that of 900 mm × 900 mm, and the average CO_2_ concentration of 2400 mm × 2400 mm was 35% lower than that of 1400 mm × 1400 mm. Tujia dwellings are located in hot summer and cold winter regions, and oversized windows lead to the loss of indoor heat in winter and the destruction of traditional architectural style. Considering the local climatic conditions and traditional architectural forms, it is suggested that the smaller window size can be adjusted to 1500 mm × 1500 mm, and the 900 mm × 900 mm window size can be appropriately increased to 1200 mm × 1200 mm to reduce the risk of indoor air pollution caused by the firepit fire behavior. This can not only help protect the local traditional residential style but can also reduce the risk of indoor air pollution caused by firepit fire behavior.

### 4.3. Windowsill Height

**Figure 24:**
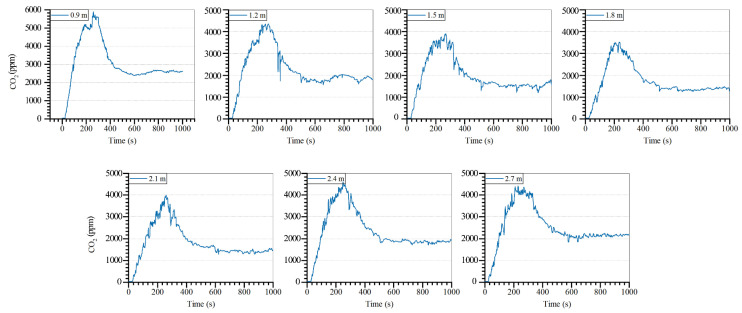
CO_2_ concentration in the firepit room with different windowsill heights.

The results of the CO_2_ concentration in the firepit for different windowsill heights are displayed in Figure 24. For all the window sizes, the indoor CO_2_ concentration rapidly increased to the peak value and subsequently decreased. With the increase in the windowsill height, the peak value increased and then decreased under all working conditions. The highest indoor CO_2_ concentrations under the seven working conditions were 5910, 4360, 3910, 3520, 4010, 4600, and 4420 ppm.

**Figure 25:**
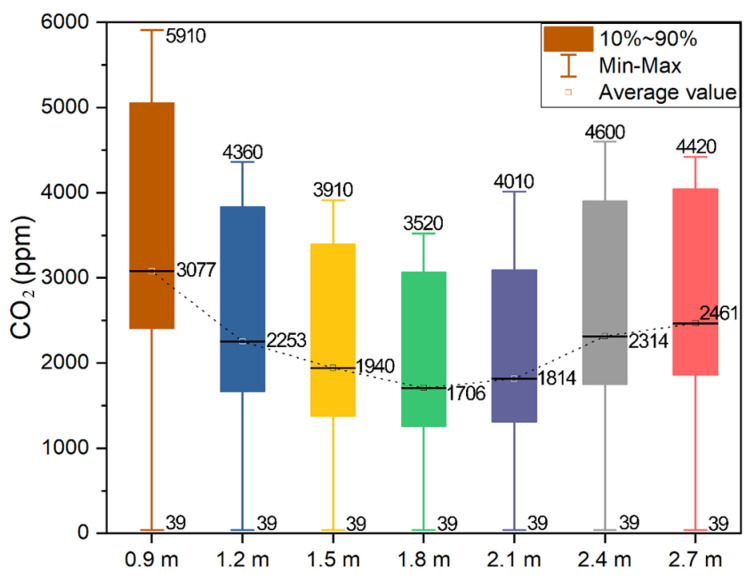
CO_2_ concentration in the firepit with various windowsill heights.

The statistical analysis results of the CO_2_ concentration in the firepit with different windowsill heights are displayed in Figure 25. For the larger window, the maximum and average CO_2_ concentrations were smaller and larger in the room. The average indoor CO_2_ concentration at 1800 mm windowsill height was 44.46%, which was 30.49% lower than that at 900 and 2700 mm, respectively. The windowsill height of 1500–1800 mm could substantially reduce the concentration of indoor pollutants and was a reasonable height. Most of the actual dwellings have a windowsill height of 900–1200 mm. However, Tujia dwellings have secondary windows that can be opened and have a windowsill height of 1800–2200 mm. In Tujia dwellings, the secondary windows can be opened to reduce the concentration of indoor pollutants during fires. In addition, the windowsill height can be adjusted to improve indoor air quality.

## 5. Discussion

This study examined the indoor-air-pollutant conditions in Hunan Tujia dwellings for reducing the risk of pollutant exposure. A large proportion of elderly people and children, who are the susceptible population, live in traditional dwellings in China. This project conducted a questionnaire study on indoor air quality in Hunan Tujia dwellings. With 34.17% participants of age > 60, indoor pollutants posed a considerable threat to the health of the elderly. The use of solid fuels (such as wood, charcoal, and coal) by Tujia residents was >90%, and was the main source of indoor pollutants. Moreover, more than half of the participants spent 8–16 h indoors daily. Prolonged exposure to the high levels of pollutants is highly detrimental to the health of residents, especially the elderly.

In this study, indoor air quality (HCHO, CO_2_, PM_2.5_, and PM_10_) was monitored in typical Tujia Zuozi and L-shaped dwellings. The results showed that indoor CO_2_, PM_2.5_, and PM_10_ concentrations were considerably affected by the fire behavior in firepits. The indoor air quality of Tujia dwellings was poor. The indoor CO_2_, PM_2.5_, and PM_10_ concentrations exceeded the values for Chinese indoor-air-quality standards. The indoor HCHO concentration in Tujia dwellings was in accordance with the Chinese standard values, and for newly renovated dwellings, the average indoor HCHO concentration (658 μg/m^3^) was cosndierably higher than this standard. The indoor formaldehyde cancer risk was 5.748 × 10^−5^ for males and 5.382 × 10^−5^ for females. The percentage of residents who never use air purifiers was >80%. The carcinogenic risk caused by formaldehyde in newly renovated residential dwellings should be further studied.

CFD software was used to simulate indoor CO_2_ dispersion when the fire was active in the firepit. The smoke diffused from the firepit to the adjacent rooms, and the most affected rooms were the master bedroom and firepit. This finding is consistent with the study results. In addition, this study proposed three strategies for controlling indoor pollutants caused by pit fires. All the rooms with passive smoke collectors in the firepit showed lower CO_2_ concentrations than the conventional rooms. The CO_2_ concentration in the firepit reduced by 43.96%. Adjusting the window size and windowsill height can considerably improve indoor air quality. The size of smaller windows should be adjusted to 1500 mm × 1500 mm, and the size of 900 mm × 900 mm windows should be increased to 1200 mm × 1200 mm, considering the preservation of the traditional residential style. The most suitable height of the windowsill was 1800 mm.

The Hunan Tujia region is one of the ten most populous minority regions in China. This study assessed the indoor pollution of Tujia dwellings, investigated the indoor pollutant dispersion patterns caused by fire bahavior, and proposed reasonable pollutant-control strategies. These strategies can help improve the indoor environment for the residents. In addition, this study provided a reference for the assessment and research of indoor pollutants in residential dwellings in other regions.

This study presents some limitations. The study proposed three mitigation strategies for the characteristics of indoor pollutants in Tujia dwellings, including the use of passive smoke collectors, adjustment of windowsill heights, and adjustment of window size. The mitigation strategies were validated using CFD software. However, these strategies were only individually validated for their effectiveness, and no multi-objective algorithms were established to obtain highly reliable mitigation strategies. In addition, the sources of indoor pollutants in Tujia dwellings are diverse, and the fire behavior of firepits is only one of the most important sources. This study addressed indoor pollution caused by the firepit fire behavior, but other sources of pollutants were not investigated. This study evaluated indoor air quality only in winter, and summer indoor pollutant profiles are different from winter indoor pollutant profiles. Future studies should focus on year-long indoor air profiles. Furthermore, these strategies were validated for indoor pollutant concentration control using software; however, their practical applications and evaluation were not studied. We will address these research deficiencies in our future study.

## 6. Conclusions

In this study, CFD was used to optimise the control of indoor pollutant concentrations in typical residential dwellings in the Hunan Tujia region. A questionnaire study on indoor air quality was conducted on Hunan Tujia residents, and indoor pollutant concentrations in typical residential dwellings were monitored to determine the potential health risks of indoor HCHO, CO_2_, PM_2.5_, and PM_10_ pollutants in residential dwellings. The dispersion of pollutant concentrations in firepits was visualised and analysed using FDS. Pollutant mitigation strategies adapted to Tujia dwellings were proposed based on respecting ethnic beliefs and traditional customs. The conclusions are summarised as follows:

- The questionnaire research results revealed that 34.17% of people participating in the research were over 60 years old, and the indoor air quality of senior citizens’ dwellings should be investigated. Solid fuels are the main source of energy for cooking and heating and are as the source of indoor pollutants in Hunan Tujia dwellings. The percentage of people opening windows all day is 74.28%, whereas more than 80% of people never use APs. Nearly 55.1% of people stay indoors for 8–16 h daily. Firepits and kitchens are rooms with the worst indoor-air-quality-assessment environment. Indoor pollutants in Hunan Tujia dwellings should be controlled in order to mitigate their effect on elderly people.
- The values of the corner house, main bedroom, hall, and second bedroom of the L-shaped dwelling were 3, 3, 3, and 3 for CO_2_, 2, 2, 2, and 1 for PM_2.5_ and 2, 2, 3, and 1 for PM_10_, respectively. The CO_2_ concentrations in the fireplace room, bedroom, hall, and kitchen of the Zuozi dwelling were 2, 2, 3, and 3, respectively; the corrected PM_2.5_ concentrations were 2, 2, 2, and 1, respectively; and the corrected PM_10_ concentrations were 2, 2, 2, and 1, respectively. The overall indoor air quality of the western Hunan Tujia dwellings was average. In newly renovated dwellings, more attention should be paid to indoor HCHO concentrations to avoid serious health threats.
- The simulation results indicated that the area above the firepit was the most dominant smoke distribution area, which is consistent with the monitoring results. We recommend the use of passive smoke collectors and adjustment of the window size and windowsill height to reduce pollutant concentrations. The simulation results of indoor pollutants with passive smoke collectors showed that the mean CO_2_ concentrations in the retrofitted bedroom, firepit, parlor, and kitchen reduced by 43.96%, 43.52%, 83%, and 118.12%, respectively, compared with that in the conventional rooms. The simulation results of firepit pollutants with different window sizes showed that the larger the window size was, the lesser the indoor air pollutants caused by firepit fire behavior. The window size of 900 mm × 900 mm should be appropriately increased to 1200 mm × 1200 mm, which does not damage the local traditional dwelling style and reduces the risk of indoor air pollution caused by firepits. The secondary windows can be opened to reduce the concentration of indoor pollutants during the fire. In addition, the windowsill height can be adjusted to improve indoor air quality.

This study has some shortcomings. Differences between the elderly, young people, and children were not considered during the indoor-air-quality assessment of Tujia dwellings. In addition, pollutant control strategies were proposed, and software simulations were used to verify their effectiveness and scientific validity, but actual engineering validation was not conducted. These shortcomings will be studied in detail in the future.

## Figures and Tables

**Table 1:** Content of the questionnaire research.

Comment	Question
Basic information	Gender, age, illness history
Odours	Oil and smoke odour, Stuffy odour, mould odour, pungent odour
Open window behaviour	Open windows all day, open windows occasionally, never open windows
Air purifier use	Never, barely use, often use, daily use
Daily staying hours	<8 h, 8–12 h, 12–16 h, >16 h
Cooking and heating energy source	Solid fuels (such as wood, charcoal, and coal), natural gas fuels, electricity
Indoor air quality evaluation	very bad, bad, moderate, good, very good

**Table 2:** Monitoring instrument parameters.

Instrument	Parameter	Accuracy	Measuring Range	Resolution
AZ-77597 Carbon dioxide analyser	CO_2_	±30 ppm or ±5% (0~5000 ppm)	0~5000 ppm	1 ppm
BR-SMART128S Air quality instrument	PM_2.5_, PM_10_	±20 μg/m^3^	0~999 μg/m^3^	1 μg/m^3^
	Formaldehyde	±30 μg/m^3^	0~3000 μg/m^3^	1 μg/m^3^

**Table 3:** Indoor-air-quality standards [57,58].

Pollutant	CO_2_	PM_2.5_	PM_10_	HCHO
Average value standard	1000 ppm	100 μg/m^3^	150 μg/m^3^	150 μg/m^3^

**Table 4:** Standard indoor-air-quality values [31,59].

CO_2_	PM_2.5_	PM_10_	Air Quality Level	Assigning Value
350~450	0~35 μg/m^3^	0~50 μg/m^3^	Best	X_1_ = 5
500~750 ppm	35~75 μg/m^3^	50~150 μg/m^3^	Better	X_1_ = 4
750~1500 ppm	75~300 μg/m^3^	150~460 μg/m^3^	Normal	X_1_ = 3
1500~2500 ppm	300~500 μg/m^3^	460~600 μg/m^3^	Worse	X_1_ = 2
>2500 ppm	>500 μg/m^3^	>600 μg/m^3^	Worst	X_1_ = 1

## Data Availability

Data available on request due to restrictions of privacy. The data presented in this study are available on request from the corresponding author. The data are no publicly available due to privacy. Please contact corresponding author before use.

## References

[1] European Commission (2003). Indoor Air Pollution: New EU Research Reveals Higher Risks than Previously Thought.

[2] Wichmann J., Lind T., Nilsson M.-M., Bellander T. (2010). PM2.5, soot and NO_2_ indoor–outdoor relationships at homes, pre-schools and schools in Stockholm, Sweden. Atmos Environ..

[3] Lelieveld J., Klingmüller K., Pozzer A., Pöschl U., Fnais M., Daiber A., Münzel T. (2019). Cardiovascular disease burden from ambient air pollution in Europe reassessed using novel hazard ratio functions. Eur. Heart J..

[4] Ostro B., Spadaro J.V., Gumy S., Mudu P., Awe Y., Forastiere F., Peters A. (2018). Assessing the recent estimates of the global burden of disease for ambient air pollution: Methodological changes and implications for low- and middle-income countries. Environ. Res..

[5] Farsani M.H., Shirmardi M., Alavi N., Maleki H., Sorooshian A., Babaei A., Asgharnia H., Marzouni M.B., Goudarzi G. (2018). Evaluation of the relationship between PM10 concentrations and heavy metals during normal and dusty days in Ahvaz, Iran. Aeolian Res..

[6] Soleimani Z., Goudarzi G., Sorooshian A., Marzouni M.B., Maleki H. (2016). Impact of Middle Eastern dust storms on indoor and outdoor composition of bioaerosol. Atmos. Environ..

[7] Surendra K.C., Takara D., Hashimoto A.G., Khanal S.K. (2014). Biogas as a sustainable energy source for developing countries: Opportunities and challenges. Renew. Sustain. Energy Rev..

[8] Chen Y., Ebenstein A., Greenstone M., Li H. (2013). Evidence on the impact of sustained exposure to air pollution on life expectancy from China’s Huai River policy. Proc. Natl. Acad. Sci. USA.

[9] Rohde R.A., Muller R.A. (2015). Air Pollution in China: Mapping of Concentrations and Sources. PLoS ONE.

[10] Moses L., Morrissey K., Sharpe R.A., Taylor T. (2019). Exposure to Indoor Mouldy Odour Increases the Risk of Asthma in Older Adults Living in Social Housing. Int. J. Environ. Res. Public Health.

[11] Chen Y., Shen H., Smith K.R., Guan D., Chen Y., Shen G., Liu J., Cheng H., Zeng E.Y., Tao S. (2018). Estimating household air pollution exposures and health impacts from space heating in rural China. Environ. Int..

[12] Ali M.U., Yu Y., Yousaf B., Munir M.A.M., Ullah S., Zheng C., Kuang X., Wong M.H. (2021). Health impacts of indoor air pollution from household solid fuel on children and women. J. Hazard. Mater..

[13] Luo Y., Zhong Y., Pang L., Zhao Y., Liang R., Zheng X. (2021). The effects of indoor air pollution from solid fuel use on cognitive function among middle-aged and older population in China. Sci. Total Environ..

[14] Shih C.-Y. (2001). Ethnicity as Policy Expedience: Clan Confucianism in Ethnic Tujia-Miao Yongshun. Asian Ethn..

[15] Wang F. (2016). “Rejoicing in Waters” Cases. Geo-Architecture and Landscape in China’s Geographic and Historic Context.

[16] Liu X., Shen G., Chen L., Qian Z., Zhang N., Chen Y., Chen Y., Cao J., Cheng H., Du W. (2021). Spatially Resolved Emission Factors to Reduce Uncertainties in Air Pollutant Emission Estimates from the Residential Sector. Environ. Sci. Technol..

[17] Du W., Wang J., Zhang S., Fu N., Yang F., Wang G., Wang Z., Mao K., Shen G., Qi M. (2021). Impacts of Chinese spring festival on household PM2.5 pollution and blood pressure of rural residents. Indoor Air.

[18] Johnson M.A., Garland C.R., Jagoe K., Edwards R., Ndemere J., Weyant C., Patel A., Kithinji J., Wasirwa E., Nguyen T. (2019). In-Home Emissions Performance of Cookstoves in Asia and Africa. Atmosphere.

[19] Yuan X., Mu R., Zuo J., Wang Q. (2015). Economic Development, Energy Consumption, and Air Pollution: A Critical Assessment in China. Hum. Ecol. Risk Assess. Int. J..

[20] Singh N., Singh S., Mall R., Verma P., Singh P., Singh R., Raghubanshi A.S. (2020). Urban ecology and human health: Implications of urban heat island, air pollution and climate change nexus. Urban Ecology.

[21] Zulauf N., Dröge J., Klingelhöfer D., Braun M., Oremek G.M., Groneberg D.A. (2019). Indoor Air Pollution in Cars: An Update on Novel Insights. Int. J. Environ. Res. Public Health.

[22] Barnes B.R. (2014). Behavioural Change, Indoor Air Pollution and Child Respiratory Health in Developing Countries: A Review. Int. J. Environ. Res. Public Health.

[23] Edwards R., Hubbard A., Khalakdina A., Pennise D., Smith K.R. (2007). Design considerations for field studies of changes in indoor air pollution due to improved stoves. Energy Sustain. Dev..

[24] Huboyo H.S., Tohno S., Lestari P., Mizohata A., Okumura M. (2014). Characteristics of indoor air pollution in rural mountainous and rural coastal communities in Indonesia. Atmos. Environ..

[25] Smith K.R., Dutta K., Chengappa C., Gusain P., Berrueta O.M.A.V., Edwards R., Bailis R., Shields K.N. (2007). Monitoring and evaluation of improved biomass cookstove programs for indoor air quality and stove performance: Conclusions from the Household Energy and Health Project. Energy Sustain. Dev..

[26] De la Sota C., Lumbreras J., Pérez N., Ealo M., Kane M., Youm I., Viana M. (2018). Indoor air pollution from biomass cookstoves in rural Senegal. Energy Sustain. Dev..

[27] Seo S.-H., Jung K.-S., Park M.-K., Kwon H.-O., Choi S.-D. (2022). Indoor air pollution of polycyclic aromatic hydrocarbons emitted by computers. Build. Environ..

[28] Zhang H., Xia Y., Cao L., Chang Q., Zhao Y. (2022). Associations between long term exposures to outdoor air pollution and indoor solid fuel use and depression in China. J. Environ. Manag..

[29] Zhu Y., Diao M., Li J. (2021). Examining indoor air pollution in a large-scale integrated transportation hub in Shanghai. Transp. Res. Part D Transp. Environ..

[30] Dubey S., Rohra H., Taneja A. (2021). Assessing effectiveness of air purifiers (HEPA) for controlling indoor particulate pollution. Heliyon.

[31] Dionova B.W., Mohammed M., Al-Zubaidi S., Yusuf E. (2020). Environment indoor air quality assessment using fuzzy inference system. ICT Express.

[32] Chen Y.-H., Tu Y.-P., Sung S.-Y., Weng W.-C., Huang H.-L., Tsai Y.I. (2022). A comprehensive analysis of the intervention of a fresh air ventilation system on indoor air quality in classrooms. Atmos. Pollut. Res..

[33] Xie R., Xu Y., Yang J., Zhang S. (2021). Indoor air quality investigation of a badminton hall in humid season through objective and subjective approaches. Sci. Total Environ..

[34] Cheng Z., Lei N., Bu Z., Sun H., Li B., Lin B. (2022). Investigations of indoor air quality for office buildings in different climate zones of China by subjective survey and field measurement. Build. Environ..

[35] He X., Zhou G., Ma Y., Li L., Fu S., Liu S., Liu C., He Y., Su Z., Liu J. (2021). Winter vacation, indoor air pollution and respiratory health among rural college students: A case study in Gansu Province, China. Build. Environ..

[36] Cai C., Sun Z., Weschler L.B., Li T., Xu W., Zhang Y. (2021). Indoor air quality in schools in Beijing: Field tests, problems and recommendations. Build. Environ..

[37] Tong Z., Chen Y., Malkawi A., Adamkiewicz G., Spengler J.D. (2016). Quantifying the impact of traffic-related air pollution on the indoor air quality of a naturally ventilated building. Environ. Int..

[38] Ruan T., Rim D. (2019). Indoor air pollution in office buildings in mega-cities: Effects of filtration efficiency and outdoor air ventilation rates. Sustain. Cities Soc..

[39] Sun Y., Wang P., Zhang Q., Ma H., Hou J., Kong X. (2015). Indoor Air Pollution and Human Perception in Public Buildings in Tianjin, China. Procedia Eng..

[40] Mannan M., Al-Ghamdi S.G. (2021). Indoor air quality in buildings: A comprehensive review on the factors influencing air pollution in residential and commercial structure. Int. J. Environ. Res. Public Health.

[41] Azuma K., Ikeda K., Kagi N., Yanagi U., Osawa H. (2018). Physicochemical risk factors for building-related symptoms in air-conditioned office buildings: Ambient particles and combined exposure to indoor air pollutants. Sci. Total Environ..

[42] Annesi-Maesano I., Baiz N., Banerjee S., Rudnai P., Rive S. (2013). Indoor Air Quality and Sources in Schools and Related Health Effects. J. Toxicol. Environ. Health Part B.

[43] Blondeau P., Iordache V., Poupard O., Genin D., Allard F. (2005). Relationship between outdoor and indoor air quality in eight French schools. Indoor Air.

[44] Madureira J., Paciência I., Rufo J., Ramos E., Barros H., Teixeira J.P., de Oliveira Fernandes E. (2015). Indoor air quality in schools and its relationship with children’s respiratory symptoms. Atmos. Environ..

[45] Bennett J., Davy P., Trompetter B., Wang Y., Pierse N., Boulic M., Phipps R., Howden-Chapman P. (2019). Sources of indoor air pollution at a New Zealand urban primary school: A case study. Atmos. Pollut. Res..

[46] Klinmalee A., Srimongkol K., Oanh N.T.K. (2009). Indoor air pollution levels in public buildings in Thailand and exposure assessment. Environ. Monit. Assess..

[47] Tran V.V., Park D., Lee Y.-C. (2020). Indoor air pollution, related human diseases, and recent trends in the control and improvement of indoor air quality. Int. J. Environ. Res. Public Health.

[48] Cincinelli A., Martellini T. (2017). Indoor Air Quality and Health. Int. J. Environ. Res. Public Health.

[49] Guo P., Yokoyama K., Piao F., Sakai K., Khalequzzaman, Kamijima M., Nakajima T., Kitamura F. (2013). Sick Building Syndrome by Indoor Air Pollution in Dalian, China. Int. J. Environ. Res. Public Health.

[50] Li W.-M., Lee S.C., Chan L.Y. (2001). Indoor air quality at nine shopping malls in Hong Kong. Sci. Total Environ..

[51] Shang Y., Li B., Baldwin A., Ding Y., Yu W., Cheng L. (2016). Investigation of indoor air quality in shopping malls during summer in Western China using subjective survey and field measurement. Build. Environ..

[52] Huai C., Xie J., Liu F., Du J., Chow D.H., Liu J. (2021). Experimental and Numerical Analysis of Fire Risk in Historic Chinese Temples: A Case in Beijing. Int. J. Arch. Herit..

[53] Liao C., Akimoto M., Bivolarova M.P., Sekhar C., Laverge J., Fan X., Lan L., Wargocki P. (2021). A survey of bedroom ventilation types and the subjective sleep quality associated with them in Danish housing. Sci. Total Environ..

[54] Afacan Y., Demirkan H. (2016). The influence of sustainable design features on indoor environmental quality satisfaction in Turkish dwellings. Arch. Sci. Rev..

[55] (2010). Industrial Standard of the People’s Republic of China: Standard for Energy Efficiency Test of Public Buildings.

[56] Hong T. (2009). A close look at the China Design Standard for Energy Efficiency of Public Buildings. Energy Build..

[57] MOHURD Announcement on Publishing National Standard ‘Code for Design of Residential Buildings for the Elderly 2016. https://www.mohurd.gov.cn/gongkai/fdzdgknr/tzgg/201702/20170227_231094.html.

[58] Zhang S.-C., Wang H., Liu Z., Zeng S., Jin Y., Baležentis T. (2019). A comprehensive evaluation of the community environment adaptability for elderly people based on the improved TOPSIS. Information.

[59] Chau C., Hui W., Tse M. (2007). Evaluation of health benefits for improving indoor air quality in workplace. Environ. Int..

[60] Cerón Bretón J.G., Cerón Bretón R.M., Martínez Morales S., Kahl J.D.W., Guarnaccia C., Lara Severino R.d.C., Rangel Marrón M., Ramírez Lara E., Espinosa Fuentes M.d.l.L., Uc Chi M.P. (2020). Health Risk Assessment of the Levels of BTEX in Ambient Air of One Urban Site Located in Leon, Guanajuato, Mexico during Two Climatic Seasons. Atmosphere.

[61] Mokhtari M., Jafari N., Mohammadi A., Hajizadeh Y., Ghanbari R., Nemati S., Abdolahnejad A. (2019). Temporal and spatial trends of airborne asbestos fiber concentrations in the urban areas of Yazd, Iran. Int. J. Environ. Sci. Technol..

[62] Nandan A., Siddiqui N.A., Kumar P. (2020). Estimation of indoor air pollutant during photocopy/printing operation: A computational fluid dynamics (CFD)-based study. Environ. Geochem. Health.

[63] Mocho P., Desauziers V., Plaisance H., Sauvat N. (2017). Improvement of the performance of a simple box model using CFD modeling to predict indoor air formaldehyde concentration. Build. Environ..

[64] Xu L., Zheng W., Xu F. (2022). Case research on kitchen fire of ancient buildings under water spray effect. Case Stud. Therm. Eng..

[65] Cai N., Chow W.-K. (2014). Numerical studies on heat release rate in a room fire burning wood and liquid fuel. Build Simul..

[66] McGrattan K., Hostikka S., McDermott R., Floyd J., Weinschenk C., Overholt K. (2013). Fire dynamics simulator technical reference guide volume 1: Mathematical model. NIST Spec. Publ..

[67] Xu L., Zheng W. (2021). Numerical simulation on the influence of low air pressure upon smoke spread and fire alarm process. Case Stud. Therm. Eng..

[68] Rovira J., Roig N., Nadal M., Schuhmacher M., Domingo J.L. (2016). Human health risks of formaldehyde indoor levels: An issue of concern. J. Environ. Sci. Health Part A.

